# The Stiffness Behavior of Asphalt Mixtures with Different Compactness under Variable Confinement

**DOI:** 10.3390/ma16020771

**Published:** 2023-01-12

**Authors:** Hancheng Dan, Penghao Yang, Wei Cao, Hongyu Shan, Zhi Zhang

**Affiliations:** 1 School of Civil Engineering, Central South University, Changsha 410075, China; 2 Power China Guiyang Engineering Co., Ltd., Guiyang 550081, China

**Keywords:** confined dynamic modulus, compactness, variable confinement, time–temperature superposition, vertical shift factor

## Abstract

The dynamic modulus is a key property determining the short- and long-term performance of asphalt pavement, and its strong dependence on confining pressure and material density (mixture compactness) has been clearly indicated in the literature. It is always challenging to reproduce three-dimensional in situ stress conditions in the laboratory. To alleviate this difficulty, in this study, a convenient experimental setup was developed, in which the lateral confinement was made present and variable as a concomitant reaction of the surrounding materials to the vertical loading. Three dense-graded mixtures were prepared to a set of four different densities and then subjected to the confined dynamic modulus test. The results indicated a significant dependence of the confined modulus on the three factors of temperature, frequency, and compactness and that the mixture with coarser gradation demonstrated a less sensitivity to these parameters. A mathematical model was developed for the dynamic modulus master curve unifying these factors by means of horizontal shifting due to the time–temperature superposition principle (validated against the variable confinement at different compactness) and the vertical shift factor as a function of reduced frequency and compactness. The adequacy of the model was demonstrated using the experimental data, and its potential application in field pavement compaction was discussed.

## 1. Introduction

Asphalt mixture is a common paving material that has been globally implemented in highway facilities, as it provides satisfactory surface roughness, riding comfort, and wear and skid resistance. This type of composite distinguishes itself from other typical civil engineering materials with the fact that it exhibits significant viscoelastic properties, owing to the presence of asphalt binder holding the aggregate particles together. The viscoelasticity-induced dependence on temperature and loading time/frequency renders the conventional use of resilient modulus insufficient in pavement structural design. In order to realistically capture the stiffness and viscoelastic features, the concept of dynamic modulus has been adopted for asphalt concrete in the more sophisticated mechanistic–empirical pavement design methodology [1,2]. Knowledge of the dynamic modulus provides solutions to stresses and strains at different depths under various temperature and loading conditions in structural analysis. It also serves as a prerequisite parameter for evaluating pavement performance such as fatigue and permanent deformation [3,4].

Given the significance of the dynamic modulus in the material and structural sense, a number of studies have been conducted to identify and understand the associated affecting factors, including temperature, loading frequency [5,6,7], air void [2,8,9], asphalt content and properties [10,11], as well as inter-particle contact and aggregate morphology [12,13], to name a few. The first three are generally considered to have major impacts on the stiffness behaviors of asphalt mixtures. The roles of temperature and frequency have been well-understood via the time–temperature superposition principle. Relative inadequacy, however, lies in the respect of void ratio or the compactness of the mixtures (the degree of compaction). Zhang et al. [2] evaluated a total of ten factors using the gray correlation analysis and pointed out that the effect of the air void was among the highest. Studies have also reported that an increase in the air void reduced the dynamic modulus for various types of mixtures such as porous asphalt concrete and epoxy-modified mixtures [8,9]. Omranian et al. [14] pointed out that the compactibility and volumetrics of asphalt mixtures were significantly impacted by the aging temperature and duration. These findings strongly suggested the necessity of quantifying the impact of compactness on the stiffness behaviors of asphalt concrete.

It is worth mentioning that the dynamic modulus test condition is restricted in the laboratory as compared to the temperature and frequency range experienced by the pavement in the field. The time–temperature superposition principle is thus invoked to shift the modulus isotherms to attain the so-called master curve, allowing a much wider temperature and frequency region to be extended, which better matches the field condition [15,16,17,18,19]. However, there is still a pitfall since the standard laboratory test is performed in the uniaxial mode, which makes the obtained one-dimensional modulus not rigorously applicable to describing the mechanistic responses of asphalt mixtures under the three-dimensional stress state in a pavement. Given this insufficiency, researchers have developed various strategies to incorporate the pressure dependence into the construction of confined dynamic moduli of asphalt mixtures, including, for instance, the use of a vertical shift factor and a variable relaxation spectrum [20,21,22,23,24]. Cao et al. [24] reported that for conventional dense-graded mixtures, the time–temperature shift factors, the relaxation spectrum, and the low-temperature stiffness were all insensitive to the confining pressure, and they proposed a triaxial characterization protocol and modeling scheme based on these observations. In theory, the triaxial dynamic modulus test provides a closer representation of the in situ stress states of pavement materials but is still faced with a deficiency due to the use of a constant confining pressure, as opposed to the variable confinement concomitant with the vertical loading in reality. Hofko [25] developed a testing suite in which water was used as the medium to apply the cyclic confining pressure (with a phase lag relative to the axial loading) to the cylinder specimen in a triaxial cell. Despite theoretical advantages, this methodology places a higher demand on the equipment and operations, which is not readily fulfilled in a typical pavement materials laboratory.

The present study devised a new yet simple-to-implement test setup that allowed us to capture the stiffness behaviors of asphalt mixtures under more realistic confining conditions at higher temperatures. The impacts of temperature, frequency, and compactness were quantified, and all three variables were considered in construction of the confined modulus master curve. The potential application of the obtained model was discussed with reference to pavement compaction under high-temperature and high-frequency conditions.

## 2. Materials and Methods

### 2.1. Materials

#### 2.1.1. Aggregate

This study considered three dense-graded asphalt mixtures denoted as AC-13, AC-20, and AC-25 with nominal maximum aggregate sizes (NMASs) of 13.2, 19, and 26.5 mm, respectively. This range of materials covered the surface to bottom asphalt layers that are typically used in highway structures in China. The basalt mineral powder and aggregate meeting the national specification JTG F40-2004 were utilized to prepare the asphalt mixtures. Table 1, Table 2 and Table 3 provide the technical indices and corresponding specification requirements.

#### 2.1.2. Asphalt Binders

Two types of asphalt binders were considered, including a #70 penetration grade neat asphalt and a styrene–butadiene–styrene (SBS) polymer-modified binder. The neat asphalt was used in AC-25 that is aimed for the bottom course, while the modified was utilized in AC-13 and AC-20 for the surface and binding courses, respectively. Both binders satisfied the criteria of the three conventional consistency indices, as shown in Table 4.

#### 2.1.3. Mixture Design

**Figure 1:**
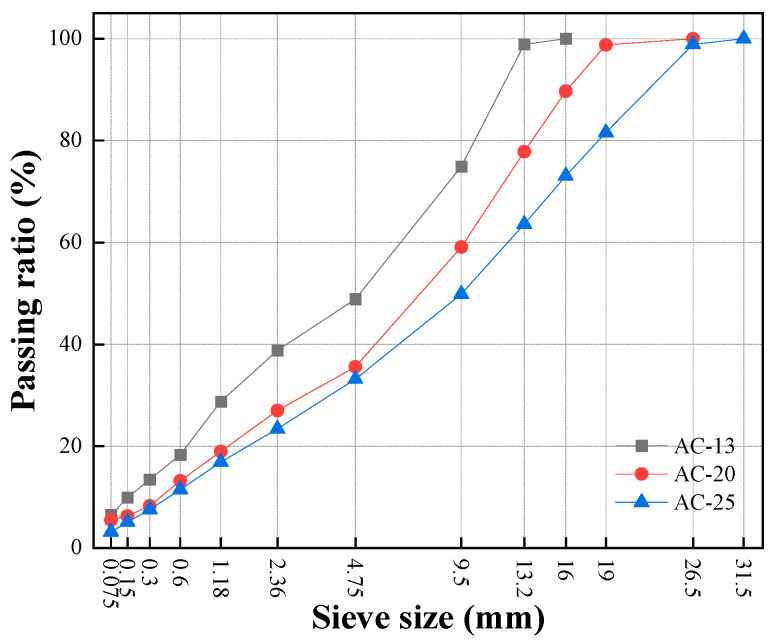
Aggregate gradation curves of the asphalt mixtures.

The aggregate gradations for the three mixtures were selected in accordance with the JTG F40-2004 specification; the resulting gradation curves are shown in Figure 1. The optimum binder contents were determined using the Marshall mix design method. Specifically, for each mixture, three levels of the asphalt–aggregate ratio (AAR) with 0.3% intervals were used to prepare the Marshall samples. A suite of design parameters was then measured, including air void, voids in mineral aggregate (VMA), voids filled with asphalt (VFA), Marshall stability, and flow number. Based on the obtained results as listed in Table 5, the optimum AARs were determined to be 4.9%, 4.3%, and 3.9% for AC-13, AC-20, and AC-25, respectively.

### 2.2. Test Sample Fabrication

**Figure 2:**
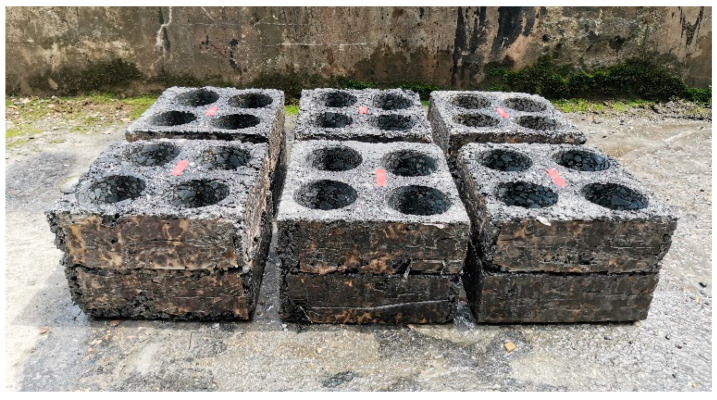
Slabs with the cylinder samples extracted from the corners.

The test specimens were fabricated using a hydraulic slab compactor to obtain the dimension of 300 × 300 × 100 mm. The challenge was how to accomplish the same set of various densities or degrees of compaction for the three mixtures with different NMASs. The essence of compaction is the aggregate particle rearrangement under external forces, and as such, a denser structure is achieved resulting in a higher strength to counteract the applied load. The compaction of asphalt mixtures is subjected to external affecting factors including the number of passes and temperature, as well as internal ones such as particle size and asphalt viscosity. With the same compaction pass, smaller particles are translated and rotated easier, and hence, the fine-graded mixtures can be compacted to the desired density with less effort. At higher compaction temperatures, the lower viscosity of the asphalt coating the aggregate facilitates the relative movement of the particles, thereby promoting the compaction process. With these considerations, we managed to achieve the same set of degrees of compaction by adjusting the compaction times and temperature for the three mixtures. That is, a relatively lower number of passes and temperatures were used for the finer mixtures, and a higher number were used for the coarser ones. After the completion of the dynamic modulus test (to be described shortly), cylinder samples of 100 mm in both diameter and height were extracted from the four corners of each slab, as illustrated in Figure 2, and used to identify the individual densities via the saturated surface dry method. The density of each slab was represented by the average of the four cylinders, as listed in Table 6. Also included are the relative densities with respect to those from the Marshall mix design and the theoretical maximum density.

### 2.3. Test Method

**Figure 3:**
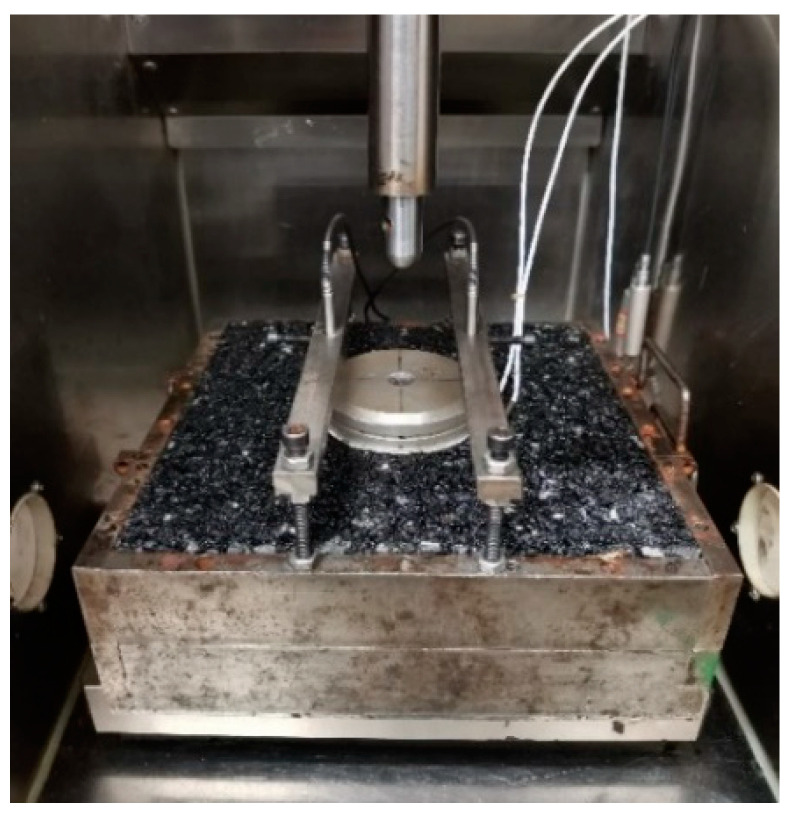
Setup of the confined dynamic modulus test.

In the standard uniaxial mode of the dynamic modulus test, the upper temperature limit is usually around 55 °C [15,16,17,18,19,26], as higher temperatures would make the testing unreliable or infeasible due to the significant presence of plastic deformation and potential specimen failure. In order for higher test temperatures and the variable confinement that is generated as a natural reaction of the surrounding materials to the vertical loading, we developed a simple yet effective test setup consisting of a slab specimen together with the mold as a whole unit placed in the environmental chamber of the universal testing machine UTM-250 kN; see Figure 3. A loading platen was placed at the center of the slab on top, and beneath it two Teflon sheets with lubricating grease in between to reduce the end friction. Two linear variable differential transducers (LVDTs) were placed in opposite directions to measure the displacement of the loading platen with respect to the mold, and the obtained data were then used to estimate the axial deformation of the materials under the platen. This configuration mimicked the in situ stress condition in which the lateral confinement is a stress reaction to and thus varies with the external loading, providing theoretical advantages over the traditional uniaxial and triaxial setup. The test was performed in a way analogous to the standard dynamic modulus test (JTG E20-2011) except for the use of relatively higher temperatures. Specifically, a frequency sweep (25, 10, 5, 1, 0.5, and 0.1 Hz) was conducted at multiple temperatures of 40 °C, 50 °C, 60 °C, and 70 °C. In order to minimize potential damage and permanent deformation accumulation, the loading was executed from low to high temperatures and from high to low frequencies. A time interval of 2 min was applied between two consecutive frequencies. The load levels were determined through trial and error to obtain an axial deformation in the range of 50 to 100 microstrains such that the material response was dominated by linear viscoelasticity. Two replicates were used for each combination of compactness and mixture, and the relative differences in the dynamic modulus were within 20%, comparable to the conventional unconfined dynamic modulus test.

## 3. Results and Discussion

This section presents the results from the confined modulus test and discusses the effects of the three predominant affecting factors of temperature, frequency, and compactness. The explorative effort is then described as to the construction of the modulus master curve as a function of these factors.

### 3.1. The Affecting Factors

#### 3.1.1. Effect of Temperature

**Figure 4:**
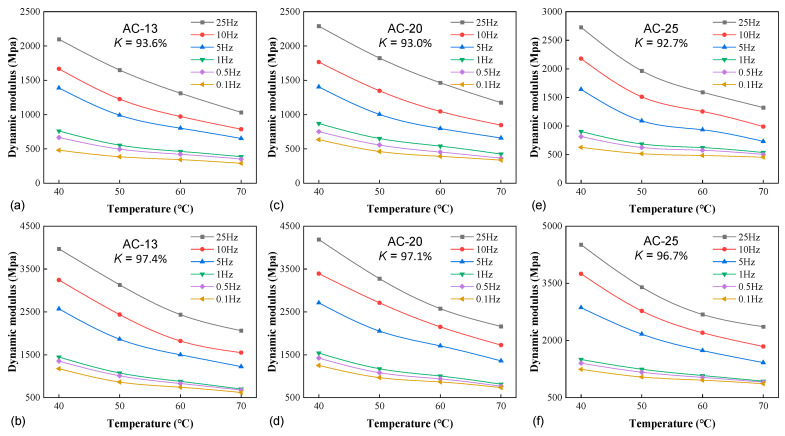
The effect of temperature: (**a**) AC-13 (*K* = 93.6%), (**b**) AC-13 (*K* = 97.4%), (**c**) AC-20 (*K* = 93.0%), (**d**) AC-20 (*K* = 97.1%), (**e**) AC-25 (*K* = 92.7%), and (**f**) AC-25 (*K* = 96.7%).

Figure 4 illustrates the impact of temperature on the confined stiffness of the three mixtures. For conciseness, only the lowest and highest degrees of compaction were provided for each material. At a given frequency and compactness, the confined stiffness decreased with the increase in temperature, as expected. The temperature elevation lowered the viscosity of the asphalt phase, thus reducing the internal forces holding the aggregate together to resist the deformation. In order to quantify the impact of temperature, the average rate of modulus reduction with temperature, *R_T_*, is defined as

(1) $$R_{T}=\frac{\sum_{i}^{n}\frac{\left(E_{Tl}{}_{i}-E_{Th}{}_{i}\right)/E_{Tl}{}_{i}}{T_{h}-T_{l}}}{n}\times 100\%$$

where *E_T_*_h_ and *E_T_*_l_ denote, respectively, the moduli at the high (*T*_h_) and low (*T*_l_) temperatures under consideration, and the index *i* loops from 1 to *n*, the total number of modulus values.

The *R_T_* values for the temperature ranges of 40–50 °C, 50–60 °C, and 60–70 °C are 2.37%, 1.91%, and 1.73% for AC-13, 2.35%, 1.85%, and 1.67% for AC-20, and 2.26%, 1.51%, and 1.36% for AC-25, respectively. For all mixtures, the reduction rate attenuated with temperature increase as the modulus approached the lower asymptote. The same trend was also reported in an earlier study by He et al. [8] based on the standard unconfined dynamic modulus data of a dense-graded mixture. On the other hand, for all temperature ranges, *R_T_* decreased with the increase in the mixtures’ NMAS, indicating that the confined stiffness of coarser mixtures was less sensitive to temperature. As the variable confinement was not measured in the study, it was not ready to assess the dependence of *R_T_* on the confining pressure. Nevertheless, it is worth mentioning that a similar evaluation has been performed by Huang et al. [21] using the conventional triaxial dynamic modulus test (with constant confinement). They demonstrated less reduction in *R_T_* with the temperature increase for higher pressure levels. In other words, the higher confinement lowered the sensitivity of stiffness to temperature, rendering more elastic-like behavior.

#### 3.1.2. Effect of Frequency

**Figure 5:**
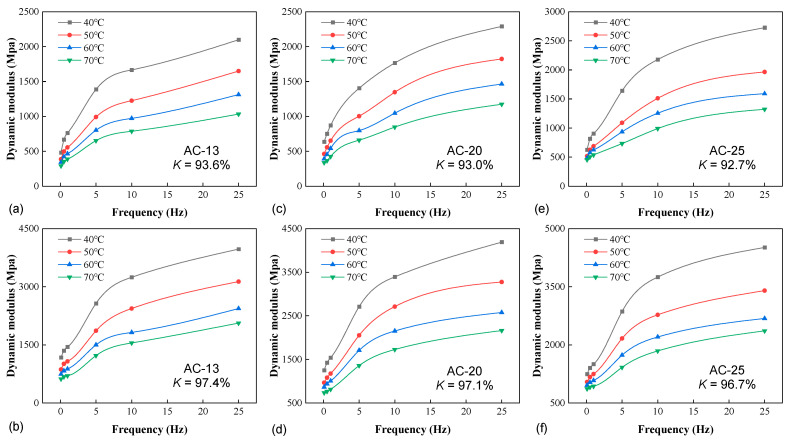
The effect of frequency: (**a**) AC-13 (*K* = 93.6%), (**b**) AC-13 (*K* = 97.4%), (**c**) AC-20 (*K* = 93.0%), (**d**) AC-20 (*K* = 97.1%), (**e**) AC-25 (*K* = 92.7%), and (**f**) AC-25 (*K* = 96.7%).

Figure 5 presents the dependence of the confined stiffness on frequency for the three mixtures at different temperatures and the two extreme degrees of compaction. As expected, the modulus in each case rose with the increasing frequency, as the elastic component grew and prevailed the response, and the overall effect was equivalent to a reduction in temperature. Similarly, the average rate of modulus increase due to frequency, *I_f_*, is defined as

(2) $$I_{f}=\frac{\sum_{i}^{n}\frac{\left(E_{fh}{}_{i}-E_{fl}{}_{i}\right)/E_{fl}{}_{i}}{f_{h}-f_{l}}}{n}\times 100\%$$

where *E_f_*_h_ and *E_f_*_l_ denote the moduli at the high (*f*_h_) and low (*f*_l_) frequencies under consideration, respectively.

The *I_f_* values for the frequency ranges of 0.1–1 Hz, 1–5 Hz, and 5–25 Hz are 32.00%, 17.70%, and 3.29% for AC-13, 27.61%, 16.08%, and 3.21% for AC-20, and 24.20%, 15.88%, and 3.14% for AC-25, respectively. For all mixtures, the increase rate decayed with increasing frequency as the moduli approached the upper asymptote. The same trend was again reported earlier by He et al. [8] based on the unconfined test. On the other hand, for all the frequency ranges, the *I_f_* values reduced with NMAS, suggesting that the confined stiffness of coarser mixtures was less sensitive to frequency.

In an unconfined test on a dense-graded Superpave-12.5 mm asphalt mixture, Ali et al. [7] showed that the average percentage of change in modulus from 25 Hz to 0.1 Hz was 80%, similar to the 72% change in the present study for AC-13 with variable confinement. Meanwhile, it is worth mentioning that using the standard triaxial test, Alamnie et al. [27] reported that an increase in the confinement reduced the sensitivity of stiffness to the loading frequency.

#### 3.1.3. Effect of Compactness

**Figure 6:**
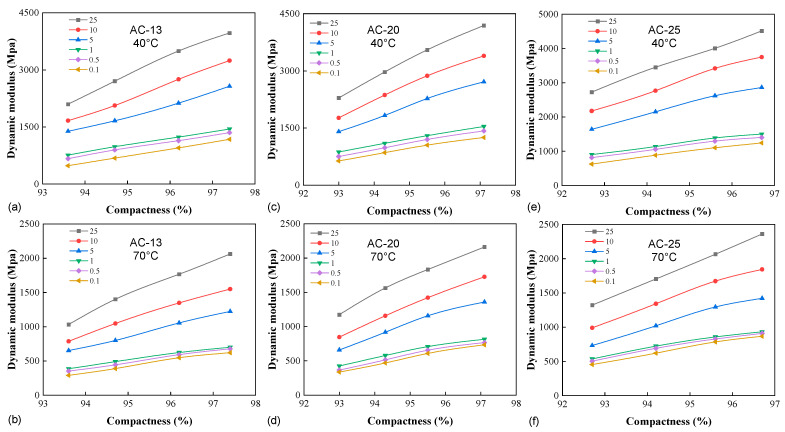
The effect of compactness: (**a**) AC-13 (40 °C), (**b**) AC-13 (70 °C), (**c**) AC-20 (40 °C), (**d**) AC-20 (70 °C), (**e**) AC-25 (40 °C), and (**f**) AC-25 (70 °C).

Figure 6 illustrates the impact of compactness on all the mixtures at different frequencies and the two extreme temperatures of 40 °C and 70 °C. In each case, a higher degree of compaction yielded a higher level of confined stiffness. An increase in the compactness is expected to yield a denser aggregate structure with more restricted particle mobility, hence the improved internal friction to resist deformation. It is also worth mentioning that higher compactness also means a lower number of voids in the mixture, leaving less room ready for crack initiation, oxidation, and moisture penetration and thus prolonged serviceability (see the increased in-place density initiative [28]). The average rate of modulus increase due to compactness, *I_K_*, is defined as

(3) $$I_{K}=\frac{\sum_{i}^{n}\frac{\left(E_{Kh}{}_{i}-E_{Kl}{}_{i}\right)/E_{Kl}{}_{i}}{K_{h}-K_{l}}}{n}\times 100\%$$

where *E_K_*_h_ and *E_K_*_l_ denote the moduli at the high (*K*_h_) and low (*K*_l_) compactness levels under consideration, respectively.

The *I_K_* values from low to high compactness levels are 28.60%, 19.31%, and 13.88% for AC-13, 26.13%, 18.76%, and 12.39% for AC-20, and 22.60%, 15.77%, and 10.02% for AC-25, respectively. For all the mixtures, the increase rate diminished with increasing compactness, presenting a saturating effect of the latter on modulus improvement. On the other hand, for all compactness ranges, the *I_K_* values reduced with the increase in NMAS, indicating that the confined stiffness of coarser mixtures was less sensitive to the degree of compaction. Further, according to these data, the addition of 1% in compactness would gain modulus increments of 20.6%, 19.1%, and 16.1% for AC-13, AC-20, and AC-25 on average, respectively. Based on the standard unconfined dynamic modulus test on a dense-graded Superpave-12.5 mm mixture, Li et al. [29] reported that 1% reduction in air void yielded 6.1% to 9.6% modulus increases. Apparently, the presence of the lateral confinement rendered a higher sensitivity of stiffness to the mixture density or compactness.

### 3.2. The Confined Modulus Master Curve

#### 3.2.1. Horizontal Shift Due to Time–Temperature Equivalence

The time–temperature superposition principle states that for a thermorheologically simple material, the modulus isotherms can be horizontally shifted to formulate a single smooth master curve, thereby greatly extending the applicable range of temperature and time/frequency. It should be noted that, however, this equivalence has been mostly validated in the cases with zero or a constant confining pressure. Its applicability should be checked against the data in the present study in the first step given the variable confinement.

The attempt to establish the modulus master curve followed the usual procedure with the use of the classic Sigmoidal function:

(4) $$\log\left(\left|E^{*}\right|\right)=\delta +\frac{\alpha }{1+e^{\beta -\gamma \log\left(f_{r}\right)}}$$

where |E^*^| is the confined dynamic modulus; *f_r_* denotes reduced frequency; *α*, *β*, *δ*, and *γ* are fitting coefficients, in which *α* is logarithmic of the lower asymptote and (*α* + *δ*) defines the upper asymptote, and *β* and *γ* are parameters related to the shape of the curve. The reduced frequency is obtained via

(5) $$f_{r}=f\times a_{T}\;\;\;\;\;\mathrm{or}\;\;\;\;\log f_{r}=\log f+\log a_{T}$$

where *f* is loading frequency, and *a_T_* is the time–temperature shift factor. A variety of forms are available in the literature to describe the functional dependence of *a_T_* on temperature [19]. This study adopted the second-order polynomial expression:

(6) $$\log a_{T}=a_{1}{\left(T-T_{R}\right)}^{2}+a_{2}\left(T-T_{R}\right)$$

where *T* is temperature, *T*_R_ denotes the reference temperature which was set to 50 °C here, and *a*_1_ and *a*_2_ are regression constants.

As above-mentioned, (*α* + *δ*) is the logarithmic of the maximum modulus corresponding to the most extreme conditions of low temperatures and/or high frequencies, for which a direct determination is not feasible in the laboratory. Considering that the stiffness of dense-graded asphalt mixtures is generally pressure-insensitive at low temperatures [24], it was decided to estimate this upper asymptote by means of the well-accredited Hirsch model which takes the volumetric parameters as the arguments [30]:

(7) $${\left|E^{*}\right|}_{\max}=P_{c}\left[4,200,000\left(1-\frac{VMA}{100}\right)+435,000\left(\frac{VFA\times VMA}{10,000}\right)\right]+\frac{1-P_{c}}{\left[\frac{\left(1-\frac{VMA}{100}\right)}{4,200,000}+\frac{VMA}{435,000\left(VFA\right)}\right]}$$

where |E^*^|_max_ is the maximum dynamic modulus, VMA and VFA are defined earlier in Table 5, and *P_c_* denote the aggregate contact volume obtained as

(8) $$P_{c}=\frac{{\left[20+\frac{435,000\left(VFA\right)}{VMA}\right]}^{0.58}}{650+{\left[\frac{435,000\left(VFA\right)}{VMA}\right]}^{0.58}}$$

**Figure 7:**
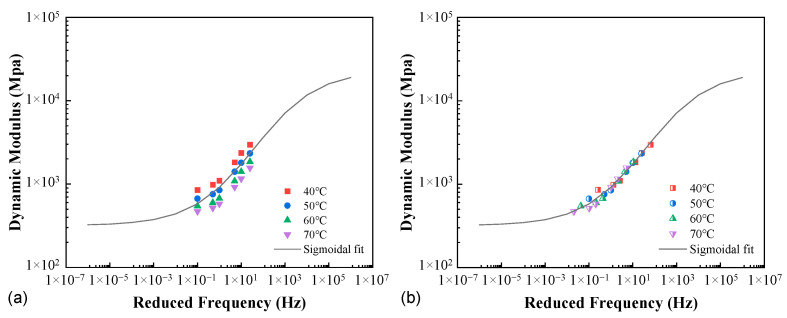
Application of the time–temperature superposition (AC-20, *K* = 94.3%, *T*_R_ = 50 °C): (**a**) before and (**b**) after the horizontal shift.

Figure 7 illustrates the application of the time–temperature superposition principle for the AC-20 mixture at the compactness *K* = 94.3%, as well as the Sigmoidal fitting curve. It appeared that at the given degree of compaction, the time–temperature equivalence was not weakened by the variable confinement. This observation needs to be checked against the other mixtures and different compactness levels. Further, the time–temperature shift factor is, in general, material-dependent. The external or internal factors that would impact the material properties in a general sense may change the time–temperature equivalence and thus enter the shift factor function as an additional argument (for instance, moisture [31]). For the interest of the present study, whether and how the compactness would affect the shift factor is the question posed. Additionally, it is worth mentioning that as a reaction to the vertical loading, the variable lateral confinement also depends on the mixture compactness.

**Figure 8:**
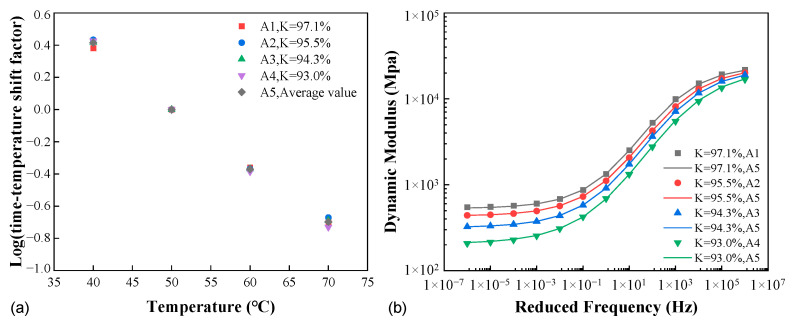
Comparisons between the individual and averaged shift factors (**a**) and the resulting dynamic modulus master curves (**b**).

To address the above concerns, the horizontal shift factors at all compactness levels for AC-20 are provided and compared in Figure 8a, represented as A1 to A4 in the legend. It is noted that the compactness within the range considered had a very minor impact, and it seemed that the shift factors could be treated as independent of the degree of compaction. For the purpose of further verification, the four sets of shift factors were averaged (denoted as A5), and the result was applied in each case of compactness to obtain the master curve, which was then compared to those using the individual shift factors. As shown in Figure 8b, the two versions of shift factors yielded very close agreement in the master curves.

The same procedure was applied to all the mixtures, and the standard error of regression (SSR) was used to quantify the degree of agreement between the measured moduli and those calculated using the individual and averaged shift factors:

(9) $$\mathrm{SSR}=\sqrt{\frac{1}{\left(n-2\right)}\sum_{i}^{n}{\left({\hat{x}}_{i}-x_{i}\right)}^{2}}$$

where *n* is the sample size (the total number of data points), and *x_i_* and ${\hat{x}}_{i}$ are the *i*-th measured and calculated moduli, respectively.

Table 7 lists the SSR results for the three mixtures. All the errors are below 5% for different compactness levels and are also comparable between the two versions of shift factors, indicating the adequacy of assuming the independence of compactness in the time–temperature shift factor. In other words, the time–temperature equivalence is mathematically not affected by the compactness within the range considered herein. Specifically, the variable *K* does not enter Equation (6) as an additional argument.

#### 3.2.2. Vertical Shift Due to Variable Compactness

It can be inferred from Figure 8a that the horizontal shifting alone cannot accommodate the strengthening effect of increase in the compactness. In order to construct the modulus master curve unifying all the affecting factors including and in addition to time and temperature, the techniques of shifting (horizontal and/or vertical) and even rotation have been proposed in the literature [32,33]. In this study, given the independence of compactness in the time–temperature horizontal shifting, it becomes reasonable to inspect its role in the vertical shifting given the strengthening effect. A number of valuable references in the literature have provided formulations of the vertical shift factor as a function of the (constant) confining pressure, moisture, or deviatoric stress in addition to time and temperature [20,34,35]. The vertical shift factor herein was expected to be a function of time, temperature, and compactness.

**Figure 9:**
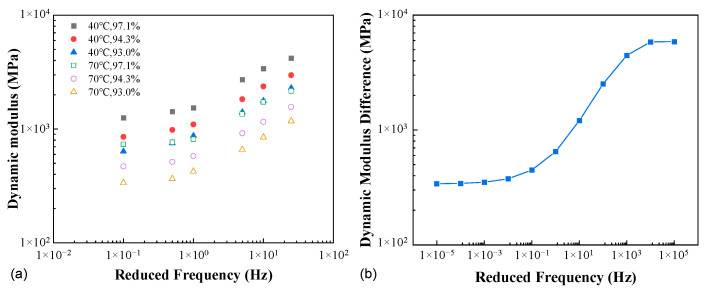
Functional dependence of the vertical shift factor (AC-20): (**a**) the modulus master curves at different compactness and temperatures, and (**b**) the modulus difference between the compactness of 97.1% and 93.0%.

Figure 9a presents the dynamic moduli for AC-20 at three compactness levels and two temperatures of 40 °C and 70 °C. For the temperature of 40 °C, the amount of vertical shift in the double logarithmic space for *K* = 97.1% is consistently higher than that for *K* = 93.0% if 94.3% is treated as the reference compactness, hence the dependence of the vertical shift factor on compactness. Further, at both temperatures, the amount of shift required varies with the reduced frequency, hence the dependence on time/frequency and temperature. In fact, the apparent dependence on reduced frequency can be illustrated more straightforwardly by the S-shaped curve in Figure 9b, i.e., the difference between the master curves corresponding to *K* = 97.1% and 93.0% (A1 and A4, respectively) in Figure 8b.

In order to couple the effects of all three factors based on the above observations, the following expression was proposed to describe the vertical shift factor:

(10) $$\log\left(\lambda \right)=\left[\delta +\frac{\alpha }{1+e^{a_{3}-a_{4}\log\left(f_{r}\right)}}\right]\left[a_{5}{\left(K-K_{0}\right)}^{2}+a_{6}\left(K-K_{0}\right)\right]$$

where *K*_0_ is the reference degree of compaction, *α*, *δ*, and *f_r_* have been defined earlier in Equation (4), and *a*_3–6_ are fitting coefficients.

The complete mathematical model for the compactness-dependent confined dynamic modulus can thus be readily written as

(11) $$\log\left(\left|E^{*}\right|\right)=\delta +\frac{\alpha }{1+e^{\beta -\gamma \log\left(f_{r}\right)}}+\left[\delta +\frac{\alpha }{1+e^{a_{3}-a_{4}\log\left(f_{r}\right)}}\right]\left[a_{5}{\left(K-K_{0}\right)}^{2}+a_{6}\left(K-K_{0}\right)\right]$$

(12) $$\log f_{r}=\log f+a_{1}{\left(T-T_{R}\right)}^{2}+a_{2}\left(T-T_{R}\right)$$

in which a total of nine parameters are to be calibrated using, for instance, the Solver function built in Microsoft Excel, recalling that (*α* + *δ*) is estimated from the Hirsch model.

**Figure 10:**
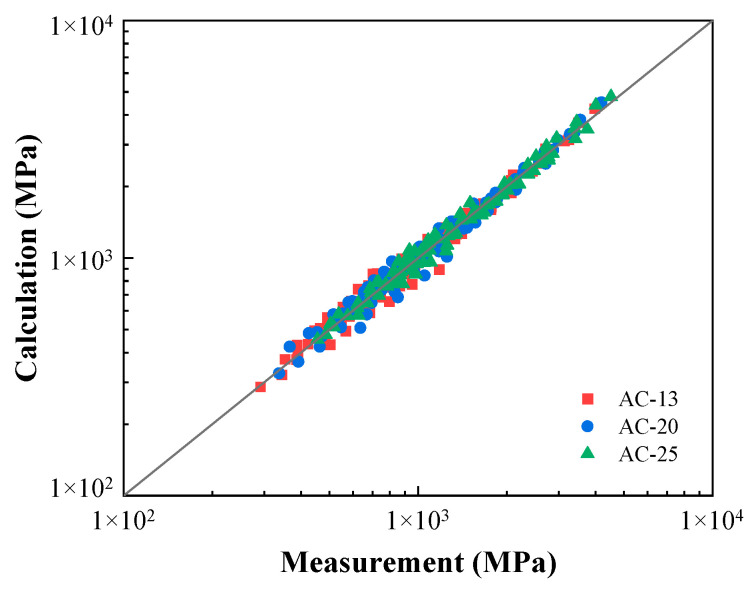
Comparison between the measured moduli and model calculations.

Table 8 summarizes the calibrated model parameters with a reference temperature of 50 °C and reference compactness of 94.7%, 94.3%, and 94.2% for AC-13, AC-20, and AC-25, respectively. Figure 10 provides a comparison of the calculated versus measured dynamic moduli for the three mixtures tested at all conditions. Excellent agreement was yielded by the model with an R-square value above 0.98. The SSR for each mixture was also identified following Equation (9), and the results are 3.73%, 3.76%, and 2.95% for AC-13, AC-20, and AC-25, respectively. The errors are within the same range of those listed in Table 7 using the individual models without the incorporation of the compactness *K*. The efficacy and reliability of the proposed model is therefore substantiated.

## 4. Potential Applications in Field Pavement Compaction

In the field compaction process, the asphalt mixtures are subjected to an extreme loading condition of high temperature and high vibrating frequency [36]. The compactness continuously increases with the number of passes, hence the modulus of the materials being compacted. Knowledge of this varying modulus under the extreme conditions would be of considerable help in advancing the intelligent compaction technique. It is highly challenging with the conventional method of the dynamic modulus test, but this difficulty could be potentially alleviated using the methodology developed in the present study.

**Figure 11:**
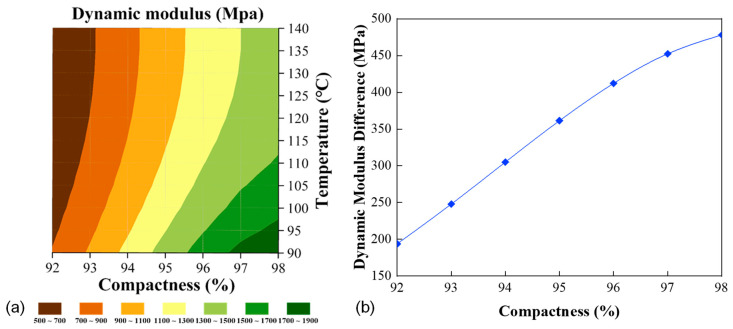
The confined moduli predicted at the field compaction conditions: (**a**) distribution of the moduli, and (**b**) difference between the moduli at the temperatures of 90 °C and 140 °C.

The surface temperature of the first compaction pass is usually around 150 °C and then decreases continuously with time [37,38]. The temperature drop yields higher viscosity in the asphalt binder, providing the mixture with more resistance to deformation through aggregate rearrangement. When the temperature drops beyond a certain point (around 80 °C), referred to as the cessation temperature, it becomes impossible to further reduce the air voids [37,38,39]. On the other hand, the typical vibrating frequency is in the range between 20 and 70 Hz [40,41,42,43]. Given this, the following discussion considers the temperature region of 90 °C to 140 °C (with a 5 °C interval) and the frequency of 50 Hz (used in the common compactor BOMAG roller BW 203 AD-4). The stiffness model calibrated for AC-20 was used to predict the confined stiffness at these extreme conditions. Figure 11a shows the distribution of the obtained modulus across the different temperatures and compactness levels. The confined stiffness varied between 518 and 1859 MPa within the temperature range considered, and the higher moduli were concentrated at the right side with higher compactness. Figure 11b provides the modulus difference between the two temperatures of 90 °C and 140 °C as a function of compactness. It is clearly shown that the effect of compactness becomes increasingly significant at higher degrees of compaction, which is consistent with the observations by Hu et al. [44] using the well-known Witczak model in estimating the dynamic modulus of asphalt mixtures for field compaction.

## 5. Conclusions

This study developed a convenient experimental setup for an asphalt mixture dynamic modulus test to more closely represent the in situ stress conditions with variable lateral confinement as a natural reaction of the surrounding materials to the vertical loading. The confined stiffness behavior was investigated at different temperatures and frequencies, and in particular, the role of mixture compactness was systematically evaluated. A mathematical model for the dynamic modulus unifying all three affecting factors was developed through the horizontal and vertical shifting technique. The following conclusions can be drawn based on the findings:

- The effects of temperature and loading frequency were consistent with those observed in the standard uniaxial dynamic modulus test. A comparison among the three mixtures evaluated indicated a less sensitivity to temperature and frequency for mixtures with a coarser gradation.
- Increase in the material compactness enhanced the confined stiffness, and the presence of the lateral confinement rendered a higher sensitivity of stiffness to compactness. The coarser gradation also exhibited a lower sensitivity of stiffness to compactness.
- With the dense-graded mixtures having different NMASs considered in this study, the time–temperature superposition principle was still applicable with the variable confinement, and further, the mathematical equivalence was not affected by the degree of compaction. The compactness was therefore not involved in horizontal shifting.
- The strengthening effect of increasing density necessitated the vertical shift factor which was described as a function of reduced frequency and compactness. The mathematical model constructed through both horizontal and vertical shifting adequately captured the dependence of the confined stiffness on time/frequency, temperature, and compactness.
- According to the model predictions for the extreme loading conditions (very high temperatures and frequencies) during field compaction, the effect of compactness became more significant at higher degrees of compaction. Further development of the model and methodology is expected such that they may potentially be incorporated in the intelligent compaction of asphalt pavement.

## Figures and Tables

**Table 1:** Technical properties of the mineral power.

Indices	Tests	Criteria	Specification
Apparent density (g/cm^3^)	2.707	≥2.5	T0352
Hydrophilic coefficient	0.7	<1	T0353
Plasticity index (%)	2	<4	T0354

**Table 2:** Technical properties of the fine aggregate.

Index	Test	Criterion	Specification
Sand equivalent (%)	75	≥60	T0334

**Table 3:** Technical properties of the coarse aggregate.

Indices	Tests					Criteria	Specification
	3–6 mm	6–10 mm	10–16 mm	16–23 mm	23–30 mm		
Flakiness index (%)		10.4	9.8	10.6	11.4	≤15	T0312
Apparent density (g/cm^3^)	2.712	2.720	2.717	2.716	2.715	≥2.6	T0304
Water absorption (%)	0.55	0.74	0.56	0.46	0.42	≤2.0	T0304
Abrasion index (%)	21.9	20.4	20.2	20.2	19.9	≤28	T0317

**Table 4:** Technical properties of the asphalt binders.

Indices	Neat	SBS Modified	Specification
Penetration, 25 °C, 100 g, 5 s (0.1 mm)	66	52	T0604
Softening point (°C)	52	73	T0606
Ductility (cm)	>100	36	T0605

**Table 5:** The Marshall mix design parameters.

Mixtures	AAR (%)	Air Void (%)	VMA (%)	VFA (%)	Marshall Stability (kN)	Flow Number (mm)
AC-13	4.9	4.4	14.4	69.5	14.07	3.3
AC-20	4.3	4.5	13.5	66.7	11.22	3.0
AC-25	3.9	4.2	12.7	67.0	14.61	2.6

**Table 6:** Density and compactness results for the slab specimens.

Mixtures	*γ*_f_ (g/cm^3^)	*γ*_f_/*γ*_fM_ (%)	*K* = *γ*_f_/*γ*_t_ (%)
AC-13	2.418	97.9	93.6
	2.448	99.1	94.7
	2.485	100.6	96.2
	2.516	101.9	97.4
AC-20	2.368	97.3	93.0
	2.402	98.7	94.3
	2.432	100.0	95.5
	2.472	101.6	97.1
AC-25	2.348	96.8	92.7
	2.384	98.3	94.2
	2.421	99.8	95.6
	2.449	100.9	96.7

Note: *γ*_f_ = saturated surface dry density, *γ*_fM_ = density from Marshall mix design, *γ*_t_ = theoretical maximum density, *K* = degree of compaction.

**Table 7:** The SSR results (%) for the use of individual and averaged shift factors.

**Mixture**	**Shift factors**	**Compactness**			
		93.6%	94.7%	96.2%	97.4%
AC-13	Individual	2.61	4.01	4.46	4.68
	Averaged	2.67	4.01	4.48	4.71
**Mixture**	**Shift factors**	**Compactness**			
		93.0%	94.3%	95.5%	97.1%
AC-20	Individual	3.83	4.10	3.94	4.46
	Averaged	3.85	4.10	3.95	4.49
**Mixture**	**Shift factors**	**Compactness**			
		92.7%	94.2%	95.6%	96.7%
AC-25	Individual	2.62	2.76	3.54	3.69
	Averaged	2.63	2.76	3.55	3.71

**Table 8:** The calibrated model parameters.

Mixtures	α	β	γ	$a_{1}$	$a_{2}$	$a_{3}$	$a_{4}$	$a_{5}$	$a_{6}$
AC-13	1.967	1.039	−0.669	0.0002	−0.039	3.477	1.248	−59.366	5.629
AC-20	1.865	1.098	−0.689	0.0002	−0.039	3.222	1.332	−51.786	4.822
AC-25	1.649	1.539	−0.911	0.0003	−0.036	−3.388	1.886	−24.026	2.166

## Data Availability

The data that support the findings will be available from the corresponding author upon reasonable request.
